# Comparison of Macular Ganglion Cell–Inner Plexiform Layer Thickness and Sectoral Ratio Asymmetry Among Different Glaucoma Types

**DOI:** 10.3390/diagnostics16070959

**Published:** 2026-03-24

**Authors:** Merve Çetin, Atılım Armağan Demirtaş, Berna Yüce, Tuncay Küsbeci

**Affiliations:** 1 Department of Ophthalmology, Izmir City Hospital, Izmir 35540, Türkiye; mervecetin254@gmail.com (M.Ç.); brnyuce@yahoo.com (B.Y.); tkusbeci@yahoo.com (T.K.); 2 Department of Ophthalmology, Izmir Faculty of Medicine, University of Health Sciences Türkiye, Izmir 35100, Türkiye

**Keywords:** ganglion cell–inner plexiform layer, primary angle-closure glaucoma, primary open-angle glaucoma, secondary open-angle glaucoma, sectoral asymmetry

## Abstract

**Background**: In this study, we aimed to evaluate and compare the diagnostic performance of peripapillary retinal nerve fiber layer (RNFL) thickness, macular ganglion cell–inner plexiform layer (GCIPL) thickness, and GCIPL asymmetry parameters in differentiating healthy eyes from primary angle-closure glaucoma (PACG), primary open-angle glaucoma (POAG), and secondary open-angle glaucoma (SOAG). **Methods**: This retrospective study included 204 eyes of 204 patients categorized into four groups: healthy controls (*n* = 46), PACG (*n* = 53), POAG (*n* = 58), and SOAG (*n* = 47). All participants underwent spectral-domain optical coherence tomography (OCT). Peripapillary RNFL thickness, sectoral and average GCIPL thickness, and GCIPL-derived asymmetry ratios were analyzed. Diagnostic performance was assessed using receiver operating characteristic (ROC) analysis. **Results**: Diagnostic accuracy varied according to glaucoma subtype. In distinguishing POAG from healthy controls, the average RNFL thickness (area under the ROC curve [AUC] = 0.82) demonstrated the highest diagnostic performance, followed by the superotemporal, inferotemporal, and average GCIPL thickness parameters. In contrast, no parameter reached an AUC of ≥0.80 in the PACG or SOAG comparisons. GCIPL asymmetry ratios exhibited limited discriminative ability across most analyses. Subtype differentiation was modest; POAG versus SOAG comparisons yielded AUC values up to 0.66, whereas PACG versus SOAG comparisons demonstrated minimal discrimination (AUC range: 0.47–0.63). **Conclusions**: Peripapillary RNFL and localized temporal GCIPL thickness measurements provide the highest diagnostic accuracy for identifying POAG. Diagnostic performance is reduced in PACG and SOAG, and the OCT parameters show limited ability to differentiate between glaucoma subtypes. GCIPL asymmetry indices do not enhance diagnostic discrimination beyond direct thickness measurements.

## 1. Introduction

Glaucoma is a progressive optic neuropathy characterized by damage to the optic nerve head (ONH), loss of the retinal nerve fiber layer (RNFL), and corresponding visual field (VF) defects. Notably, structural alterations may precede detectable functional impairment by several years [1,2,3], and VF defects become clinically detectable only after approximately 40–50% of the retinal ganglion cells have been lost [4,5]. Optical coherence tomography (OCT) has transformed the structural evaluation of glaucoma by enabling high-resolution, quantitative measurements of retinal layers [6,7,8,9,10,11]. Advances in OCT resolution have enabled the measurement of retinal layer thickness with high precision (3–6 μm). The results of previous studies have demonstrated that ganglion cell–inner plexiform layer (GCIPL) and RNFL measurements are valuable in diagnosing preperimetric glaucoma [12,13,14,15,16,17,18]. Owing to the uneven thickness of the GCIPL in each hemisphere, sector-based studies may play an important role in the early follow-up of glaucoma [19,20,21].

Several research groups have reported that evaluating superior–inferior asymmetry in macular parameters, such as total macular thickness and GCIPL thickness, is valuable for identifying early-stage glaucoma [22,23,24].

In most cases, glaucoma initially causes the loss of specific retinal ganglion cells, which may remain undetected when assessing the average GCIPL thickness [19,25,26]. Consequently, intersectoral asymmetry analysis may reveal early structural changes, even in eyes deemed clinically healthy, based on standard examinations and OCT parameters.

In this study, we compared macular GCIPL thickness, peripapillary RNFL thickness, and sectoral ratio asymmetry in patients with different glaucoma types and a healthy control group using OCT and evaluated their diagnostic performance in distinguishing early-stage glaucomatous eyes from healthy individuals. We aimed to identify OCT-derived biomarkers that could facilitate the early detection of macular involvement in glaucoma and clinical diagnosis by analyzing both global and sectoral parameters. The use of sectoral ratios may enable the more precise detection of focal glaucomatous damage while minimizing the effects of anatomical variations.

## 2. Materials and Methods

### 2.1. Study Design and Participants

This retrospective cross-sectional study was conducted at a single tertiary referral center. All examinations were performed in accordance with the Declaration of Helsinki and were approved by the Institutional Review Board and Ethics Committee of Izmir City Hospital.

A total of 204 eyes of 204 participants were included and classified into four groups according to their clinical diagnosis: healthy controls (*n* = 46), primary angle-closure glaucoma (PACG; *n* = 53), primary open-angle glaucoma (POAG; *n* = 58), and secondary open-angle glaucoma (SOAG; *n* = 47).

Glaucoma diagnoses were established according to the criteria defined in the European Glaucoma Society guidelines [27]. Subjects with a confirmed diagnosis of early-stage PACG, POAG, or SOAG based on clinical criteria, including a mean deviation on visual field testing better than –6 dB, were included.

The PACG group included eyes with glaucomatous optic neuropathy and corresponding visual field defects in the presence of occludable or closed anterior chamber angles, confirmed by means of gonioscopy.

The POAG group included eyes with open anterior chamber angles and characteristic glaucomatous optic disc changes accompanied by corresponding VF defects, irrespective of the IOP level.

The SOAG group included eyes with open anterior chamber angles and glaucomatous optic neuropathy with corresponding VF defects secondary to an identifiable ocular or systemic cause (pseudoexfoliation, pigment dispersion, uveitic glaucoma, or steroid-induced glaucoma). All cases in the SOAG group demonstrated a documented secondary mechanism responsible for the elevated IOP and optic nerve damage.

The healthy control group included subjects with normal optic disc appearance, normal VF results on standard automated perimetry (SAP), intraocular pressure (IOP) < 22 mmHg, no history of elevated IOP, and no evidence of RNFL defects on fundus examination.

### 2.2. Ophthalmic Examination

All participants underwent a comprehensive ophthalmic examination, which included the following: measurement of best-corrected visual acuity (BCVA), refraction, intraocular pressure (IOP; measured using Goldmann applanation tonometry), slit-lamp biomicroscopy, gonioscopic evaluation of the anterior chamber angle, and dilated fundus examination.

### 2.3. Inclusion and Exclusion Criteria

The inclusion criteria for all participants were as follows: a BCVA of 20/40 or better; a spherical equivalent refractive error within ±6.00 diopters; an astigmatism within ±3.00 diopters; and good-quality OCT images with a quality index of 6/10 or higher and free of motion or segmentation artifacts.

Subjects with a history of intraocular surgery other than uncomplicated cataract surgery or ocular diseases that could affect the retina or optic nerve were excluded. Eyes with poor-quality OCT images or segmentation errors were also excluded.

### 2.4. OCT Imaging

OCT imaging was performed using an Optopol Revo 60 spectral-domain (SD-OCT) system (Optopol Technology, Zawiercie, Poland) equipped with software version 11.5.0. The system operates at a scan speed of 60,000 A-scans/s and offers an axial resolution of 5 μm, thereby enabling high-resolution visualization of posterior ocular structures. We obtained two scan protocols for OCT imaging: a 7 × 7 mm macular scan centered on the fovea in the retina mode and a 6 × 6 mm peripapillary scan centered on the ONH, following a 3.40 mm diameter circle in the disc mode.

**Figure 1:**
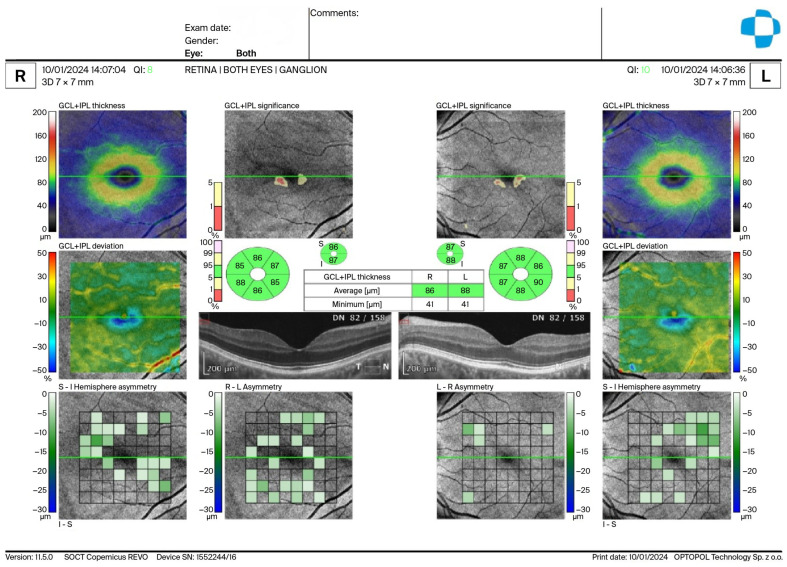
Representative macular ganglion cell–inner plexiform layer (GCIPL) thickness map obtained by automatic seg-mentation using the device software. Thickness values are shown for the average GCIPL, minimum GCIPL, and six parafoveal sectors: superior (S), inferior (I), superonasal (SN), inferonasal (IN), superotemporal (ST), and in-ferotemporal (IT).

Macular GCIPL was automatically segmented by the device software, and thickness values were obtained for the average GCIPL, minimum GCIPL, and six parafoveal sectors, including superior (S), inferior (I), superonasal (SN), inferonasal (IN), superotemporal (ST), and inferotemporal (IT) regions (Figure 1).

Sectoral GCIPL thickness values were analyzed to evaluate regional macular involvement and asymmetry. The hemispherical GCIPL thickness was calculated by summing the thickness values of the superior (S, SN, and ST sectors) and inferior (I, IN, and IT sectors) hemispheres. In addition to hemispheric comparisons, intersectoral GCIPL asymmetry was evaluated using predefined sectoral ratios, including S/I, SN/IN, ST/IT, SN/IT, ST/IN, temporal-to-nasal (ST + IT/SN + IN), and superior-to-inferior (S + SN + ST/I + IN + IT) ratios.

These ratios were calculated to assess localized ganglion cell asymmetry and to determine their diagnostic performance in distinguishing between glaucomatous and healthy eyes, in addition to the early detection of glaucomatous damage in eyes classified as clinically healthy.

### 2.5. Statistical Analysis

All statistical analyses were performed using R software (version 4.4.1; R Core Team, Vienna, Austria). Descriptive statistics are presented as mean (standard error [SE]) for continuous variables and as frequency and percentage (*n*, %) for categorical variables.

Comparisons of continuous variables among the four study groups (healthy controls, PACG, POAG, and SOAG) were performed using analysis of covariance (ANCOVA) to control for potential confounding factors (e.g., age and sex); the results are reported as adjusted means. For baseline demographic variables that did not require covariate adjustment, one-way analysis of variance (ANOVA) was applied. When a significant overall group effect was detected, post hoc pairwise comparisons were conducted using Tukey’s honest significant difference test, considering variance homogeneity.

Categorical variables were compared using Pearson’s chi-squared test. When Cochran’s assumptions were violated (i.e., >20% of expected cell counts < 5 or any expected count < 1), Fisher’s exact test was used. Monte Carlo simulation was applied for larger contingency tables when appropriate.

To evaluate the discriminative ability of OCT macular and optic disc parameters in differentiating glaucoma subtypes and healthy eyes, receiver operating characteristic (ROC) curve analysis was performed [28,29]. The areas under the ROC curve (AUCs) with corresponding 95% confidence intervals (95% CI) were calculated for each parameter. Optimal cut-off values were determined using the Youden index, which maximizes the sum of sensitivity and specificity.

A two-tailed *p*-value of <0.05 was considered statistically significant for all analyses.

## 3. Results

### 3.1. Baseline Demographic and Clinical Characteristics

Table 1 presents the basic demographic and clinical information for the four study groups: healthy controls, PACG, POAG, and SOAG.

A significant difference was recorded in the average age between the groups (*p* < 0.001). Post hoc analysis results revealed that the mean age was significantly elevated in the POAG (69.64 ± 0.99 years) and SOAG (67.23 ± 1.10 years) groups compared to the control (62.93 ± 1.11 years) and PACG (62.40 ± 1.03 years) groups (*p* < 0.05).

A significant difference was recorded in sex distribution between the groups (*p* = 0.015), with a higher percentage of male participants (66%) in the SOAG group than in the other groups. No notable difference was observed in laterality (right/left eye distribution) between the groups (*p* = 0.999).

BCVA was significantly different between the groups (*p* = 0.003); however, IOP exhibited no significant difference between the groups (*p* = 0.112). A significant difference was recorded in central corneal thickness between the groups (*p* = 0.015).

### 3.2. Peripapillary RNFL Parameter

The peripapillary RNFL parameters for all study groups are presented in Table 2. A statistically significant difference was recorded in the average peripapillary RNFL thickness among the four groups (*p* = 0.001). The healthy group exhibited the highest mean thickness (98.31 ± 2.04 µm); in comparison, the POAG group exhibited the most pronounced thinning (86.98 ± 1.84 µm).

### 3.3. Macular GCIPL Parameters

The detailed comparisons of macular GCIPL thickness are summarized in Table 2. Statistically significant differences in sectoral GCIPL thickness were observed between the groups. The POAG group consistently exhibited significantly lower values than the control group across multiple sectors: superior (*p* = 0.007), inferior (*p* = 0.013), inferonasal (*p* = 0.023), and superonasal (*p* = 0.046). Notably, the temporal sectors (ST and IT) exhibited significantly lower values (both *p* < 0.001).

The average GCIPL thickness differed significantly between the groups (*p* = 0.008), with the lowest value recorded in the POAG group (78.88 ± 1.51 µm). In contrast, the minimum GCIPL thickness did not reach statistical significance between the groups (*p* = 0.224).

Regarding GCIPL asymmetry, most ratio-based parameters, including S/I, SN/IN, ST/IT, ST/IN, and the hemispheric ratio (S + SN + ST/I + IN + IT), exhibited no significant differences (*p* > 0.05). However, specific indices, such as the SN/IT ratio (*p* = 0.025) and temporal-to-nasal ratio (ST + IT/SN + IN; *p* = 0.025), exhibited significant differences, suggesting a disproportionate involvement of the temporal macula in certain glaucoma subtypes.

### 3.4. ROC Analysis

ROC curve analysis was performed to evaluate the diagnostic performance of OCT-derived parameters in differentiating glaucoma subtypes from healthy eyes.

#### 3.4.1. POAG vs. Healthy Controls

When distinguishing between POAG and healthy eyes, the average peripapillary RNFL thickness demonstrated the highest level of diagnostic accuracy (AUC = 0.82; 95% CI: 0.73–0.90). At an optimal cut-off value of 89.5 µm, the sensitivity was 65.5%, and the specificity was 93%.

Among the macular parameters, the average GCIPL thickness (AUC = 0.80; 95% CI: 0.71–0.88), superotemporal GCIPL (AUC = 0.80; 95% CI: 0.72–0.89), and inferotemporal GCIPL (AUC = 0.79; 95% CI: 0.70–0.88) demonstrated good discriminatory ability. Notably, the inferotemporal sector demonstrated high specificity (91.3%) at its optimal threshold.

In contrast, GCIPL asymmetry ratios exhibited limited diagnostic value, with AUC values generally <0.70.

#### 3.4.2. PACG vs. Healthy Controls

For PACG versus controls, none of the evaluated parameters exhibited an AUC ≥ 0.80. The SN/IT GCIPL ratio exhibited the highest diagnostic performance (AUC = 0.69; 95% CI: 0.59–0.80). At the optimal cut-off value of 1.06, the sensitivity and specificity were 60.4% and 73.9%, respectively. Overall, the discriminative performance of PACG was moderate to weak.

#### 3.4.3. SOAG vs. Healthy Controls

Similarly, in the SOAG versus control group, no parameter exhibited an AUC of at least 0.80. The average RNFL thickness demonstrated the greatest effectiveness (AUC = 0.69; 95% CI: 0.58–0.80). At a cut-off value of 87.5 µm, the sensitivity was 44.4%, whereas the specificity was high at 95.3%, indicating good confirmatory capability but limited screening ability.

#### 3.4.4. POAG vs. PACG

Diagnostic performance was limited in differentiating POAG from PACG (AUC range: 0.52–0.69). Average RNFL thickness exhibited the highest AUC (0.69; 95% CI: 0.58–0.79), with 70.9% sensitivity and 64.7% specificity at a threshold of 92.5 µm.

Among the macular parameters, the superotemporal and superior GCIPLs exhibited relatively better performance (AUC = 0.68 and 0.67, respectively). However, no parameter demonstrated strong discriminatory ability. The results of the ROC analysis of the OCT parameters used to differentiate between POAG and PACG are shown in Table 3.

#### 3.4.5. POAG vs. SOAG

The discriminative performance of distinguishing POAG from SOAG was weak (AUC range: 0.57–0.66). The highest AUC values were observed in the inferior and superotemporal GCIPL sectors (both AUC = 0.66). The average RNFL thickness, which exhibited strong performance in distinguishing POAG from controls, demonstrated reduced diagnostic value in this comparison. This finding indicates substantial structural overlap between the two open-angle glaucoma subtypes. The results of the ROC analysis of the OCT parameters used to differentiate between POAG and SOAG are shown in Table 4.

#### 3.4.6. PACG vs. SOAG

The discriminative performance was poor, with AUC values ranging from 0.47 to 0.63. Most parameters exhibited values close to 0.50, suggesting that the two glaucoma subtypes demonstrated significant structural overlap and that the discrimination was almost random.

Notably, the asymmetry (ratio-based) parameters exhibited superior performance compared to the absolute thickness measurements. The superotemporal-to-inferotemporal GCIPL ratio yielded the highest AUC (0.63). At the optimal cut-off value of 0.98, these parameters demonstrated a sensitivity of 66% and a specificity of 59.6%. The results of the ROC analysis of the OCT parameters used to differentiate between PACG and SOAG are shown in Table 5.

## 4. Discussion

In this study, we comprehensively assessed the diagnostic effectiveness of peripapillary RNFL thickness, macular GCIPL thickness, and GCIPL-related asymmetry parameters in differentiating healthy eyes from those affected by POAG, PACG, and SOAG, in addition to distinguishing between various glaucoma subtypes. Our findings indicate that diagnostic accuracy is considerably influenced by subtype and that absolute thickness measurements are more accurate than asymmetry-based indices.

The POAG group exhibited the highest diagnostic performance. Average peripapillary RNFL thickness demonstrated the strongest discriminatory ability, followed by superotemporal, inferotemporal, and average GCIPL thickness parameters. These results suggest that both peripapillary and localized macular structural measurements offer reliable discrimination in POAG. These findings are consistent with those of previous studies demonstrating the superior diagnostic performance of peripapillary RNFL thickness and RNFL volume measurements in early glaucoma detection [30,31,32].

Temporal macular sectors are particularly susceptible to glaucomatous optic neuropathy. Consistent with our findings, Chen et al. identified inferotemporal GCIPL thickness as the most accurate macular parameter for preperimetric glaucoma [19]. This finding suggests that localized temporal GCIPL thinning is a reliable structural indicator of glaucomatous damage.

The diagnostic performance for PACG and SOAG, in comparison, was much lower. No parameter in these subgroups exhibited an AUC ≥ 0.80. Although the selected sectoral GCIPL parameters demonstrated moderate specificity, the overall discriminatory ability remained limited. This finding suggests that structural OCT parameters may be more effective in detecting the characteristic damage pattern of POAG than in other glaucoma subtypes. In SOAG, the diversity of the underlying etiologies may lead to inconsistent macular involvement, consequently diminishing ROC performance.

The discriminative performance of the sectorial analysis was limited when POAG was compared with SOAG. Although the inferior and superotemporal GCIPL thickness parameters yielded the highest AUC values, the overall diagnostic separation remained limited. Notably, average RNFL thickness, which was highly effective in distinguishing POAG from healthy controls, was less effective in differentiating POAG from SOAG.

Distinguishing between PACG and SOAG is particularly challenging. Most parameters demonstrated AUC values between 0.47 and 0.63, indicating minimal discriminatory capacity. Notably, the superotemporal-to-inferotemporal ratio exhibited relatively higher AUC values in this comparison; however, the overall performance remained limited from a clinical perspective. These results imply that macular sectorial analysis in non-POAG subtypes may substantially overlap, which restricts the ability to classify the subtypes using OCT. This finding may be partially explained by the heterogeneity of the SOAG group, encompassing various etiologies, such as pseudoexfoliation, pigment dispersion, uveitic glaucoma, and steroid-induced glaucoma. These conditions may exhibit unique patterns of structural damage and progression, resulting in heightened variability in OCT measurements and diminished diagnostic consistency.

Although previous studies have reported the superior sensitivity of minimum GCIPL thickness for early glaucoma detection [9], our findings did not indicate a specific diagnostic benefit for this parameter. The minimum GCIPL showed only moderate or poor performance in the subgroup analyses, particularly in subtype differentiation. This observation is consistent with that of Chen et al., who observed no clear superiority of minimum GCIPL over other structural measures [19]. The differences across studies may be partly explained by differences in study design, disease stage distribution, and segmentation algorithms.

The GCIPL asymmetry ratios demonstrated limited diagnostic utility across most comparisons. In differentiating glaucomatous eyes from healthy eyes, the AUC values for the ratio-based indices generally approached the level of chance. Furthermore, the asymmetry parameters failed to meaningfully differentiate between glaucoma subtypes.

These findings contrast with those of Takemoto et al., who demonstrated high diagnostic accuracy of asymmetry metrics in preperimetric glaucoma [33]. Sharifipour et al. and Park et al. also reported improved performance of specific asymmetry indices in early disease stages [34,35]. Hwang et al. demonstrated that asymmetry parameters perform best in early-to-moderate glaucoma but exhibit reduced utility in other stages [22]. In a number of studies on early or preperimetric glaucoma, researchers have used customized asymmetry algorithms. Our cohort included multiple glaucoma subtypes with heterogeneous structural patterns, potentially reducing the stability and generalizability of ratio-based parameters in clinical populations.

Overall, our findings suggest that direct sectoral and average thickness measurements, particularly those of the average RNFL thickness and temporal GCIPL sectors, are the most reliable structural markers derived from OCT for the assessment of glaucoma. ROC analysis consistently favored absolute thickness parameters over asymmetry-based ratios. Although OCT measurements effectively distinguish POAG from healthy eyes, they have limitations in differentiating between glaucoma subtypes.

This study has several methodological limitations. First, it lacks visual field correlation, as no structure–function analysis was performed to relate OCT-derived GCIPL and RNFL thinning to corresponding visual field sensitivity changes, limiting its clinical relevance. Second, although participants were categorized as having early-stage glaucoma, there was no further stratification by severity within this range, which may obscure differences in asymmetry parameters between preperimetric and early perimetric disease. Third, the exclusive use of the Optopol Revo 60 SD-OCT restricts generalizability because OCT measurements are not interchangeable across platforms; including other devices such as Cirrus or Spectralis would improve external validity. Fourth, the relatively small control group (*n* = 46) reduces statistical power and may compromise the specificity estimates in the ROC analyses.

The authors of future prospective studies could evaluate macular GCIPL thickness and sectoral asymmetry patterns across different glaucoma subtypes, integrated with the Glaucoma Risk Calculator as a targeted screening tool [36]. Community- or clinic-based participants over age 40 would first be screened using the calculator, which demonstrates high sensitivity (93.5%) and specificity (91.3%), through handheld tonometry and pachymetry, and high-risk individuals would then undergo SD-OCT imaging. Detailed macular GCIPL analysis, including average, minimum, and sectoral thickness measurements and vertical asymmetry indices, would enable comparison of structural patterns among patients with POAG, normal-tension glaucoma (NTG), pseudoexfoliation glaucoma (PXG), and pigmentary glaucoma (PDG) relative to age-matched low-risk controls.

This design is valuable because glaucoma subtypes exhibit distinct OCT profiles: POAG and NTG exhibit inferotemporal loss, PDG tends toward superior-nasal thinning, and PXG demonstrates greater inter-eye asymmetry and faster GCIPL progression. Researchers could investigate whether superior–inferior GCIPL asymmetry patterns differ systematically across subtypes and whether combining local asymmetry indices with minimum GCIPL thickness improves diagnostic performance. Particular focus should be placed on temporal macular sectors, which consistently show the strongest discrimination of early glaucomatous change, enabling earlier detection of preperimetric disease in high-risk patients identified through risk-based screening. The protocol may also aid in identifying patients with early angle-closure glaucoma, additionally triggering earlier gonioscopy.

## 5. Conclusions

Peripapillary RNFL and localized macular ganglion GCIPL thickness parameters, particularly average RNFL, superotemporal GCIPL, and inferotemporal GCIPL, provided the highest diagnostic accuracy for identifying POAG. In contrast, the diagnostic performance was weaker in PACG and SOAG, and differentiating between subtypes using OCT parameters remained challenging. GCIPL asymmetry ratios did not provide any additional diagnostic benefit. These findings emphasize the importance of direct thickness measurements in the structural assessment of glaucoma.

## Figures and Tables

**Table 1:** Baseline demographic and clinical characteristics of the study groups.

	Healthy *n* = 46	PACG *n* = 53	POAG *n* = 58	SOAG *n* = 47	*p*	*p*1	*p*2	*p*3	*p*4	*p*5	*p*6
	Mean (SE) or n (%)										
Age (years)	62.93 (1.11)	62.4 (1.03)	69.64 (0.99)	67.23 (1.1)	**<0.001**	0.985	**<0.001**	**0.033**	**<0.001**	**0.008**	0.366
Gender (M/F)	17 (37%)/29 (63%)	22 (41.5%)/31 (58.5%)	33 (56.9%)/25 (43.1%)	31 (66%)/16 (34%)	**0.015**	0.798	0.068	**0.010**	0.153	**0.025**	0.456
Side (R/L)	22 (47.8%)/24 (52.2%)	26 (49.1%)/27 (50.9%)	28 (48.3%)/30 (51.7%)	23 (48.9%)/24 (51.1%)	0.999	1	1	1	1	1	1
BCVA (Snellen)	0.93 (0.04)	0.75 (0.05)	0.74 (0.04)	0.85 (0.05)	**0.003**	**0.009**	**0.006**	0.318	0.997	0.560	0.392
IOP (mm Hg)	16.29 (0.66)	18.46 (0.62)	17.63 (0.6)	17.52 (0.65)	0.112	0.071	0.461	0.563	0.786	0.729	0.999
CCT (µm)	548.34 (6.83)	552.68 (6.11)	527.33 (5.9)	549.34 (6.3)	**0.015**	0.963	0.106	1.000	**0.021**	0.982	0.050

PACG: Primary angle-closure glaucoma; POAG: Primary open-angle glaucoma; SOAG: Secondary open-angle glaucoma; SE: Spherical error; M: Male; F: Female; R: Right; L: Left; BCVA: Best corrected visual acuity; IOP: Intraocular pressure; CCT: Central corneal thickness. *p*1 (Healthy vs. PACG); *p*2 (Healthy vs. POAG); *p*3 (Healthy vs. SOAG); *p*4 (PACG vs. POAG); *p*5 (PACG vs. SOAG); *p*6 (POAG vs. SOAG). Bold *p*-values indicate statistical significance (*p* < 0.05).

**Table 2:** Comparison of peripapillary RNFL, macular GCIPL thickness, and GCIPL asymmetry parameters among the study groups.

	Healthy *n* = 46	PACG *n* = 53	POAG *n* = 58	SOAG *n* = 47	*p*	*p*1	*p*2	*p*3	*p*4	*p*5	*p*6
	Adjusted mean (SE)										
Average RNFL (µm)	98.31 (2.04)	92.43 (1.89)	86.98 (1.84)	91.28 (1.98)	**0.001**	0.137	**<0.001**	0.075	0.188	0.976	0.368
GCIPL S (µm)	87.34 (1.63)	83.29 (1.54)	79.41 (1.53)	84.18 (1.6)	**0.007**	0.253	**0.004**	0.527	0.313	0.979	0.126
GCIPL I (µm)	87.4 (1.91)	81.58 (1.79)	79.26 (1.79)	84.41 (1.87)	**0.013**	0.106	**0.015**	0.690	0.810	0.706	0.181
GCIPL IN (µm)	88 (1.69)	82.66 (1.58)	81.14 (1.58)	84.52 (1.66)	**0.023**	**0.087**	**0.023**	0.472	0.913	0.854	0.436
GCIPL SN (µm)	87.87 (1.53)	84.42 (1.44)	81.79 (1.44)	84.86 (1.51)	**0.046**	0.335	**0.028**	0.516	0.596	0.997	0.436
GCIPL ST (µm)	84.21 (1.69)	79.31 (1.58)	74.71 (1.58)	80.64 (1.66)	**<0.001**	0.136	**<0.001**	0.450	0.195	0.940	0.044
GCIPL IT (µm)	86.03 (1.81)	78.1 (1.7)	76.16 (1.69)	81.92 (1.78)	**<0.001**	**0.007**	**<0.001**	0.385	0.863	0.419	0.081
Average GCIPL (µm)	86.65 (1.61)	81.7 (1.51)	78.88 (1.51)	82.57 (1.59)	**0.008**	0.137	**<0.001**	0.075	0.188	0.976	0.368
Minimum GCIPL (µm)	42.95 (1.75)	39.34 (1.64)	38.52 (1.64)	41.56 (1.76)	0.224	0.415	0.278	0.946	0.986	0.802	0.568
GCIPL S/I	1 (0.02)	1.04 (0.02)	1.03 (0.02)	1 (0.02)	0.605	0.670	0.899	1.000	0.979	0.714	0.898
GCIPL SN/IN	1 (0.01)	1.03 (0.01)	1.02 (0.01)	1.01 (0.01)	0.542	0.500	0.882	0.982	0.929	0.765	0.981
GCIPL ST/IT	0.98 (0.02)	1.03 (0.02)	1 (0.02)	0.99 (0.02)	0.303	0.290	0.952	0.993	0.637	0.478	0.993
GCIPL SN/IT	1.03 (0.02)	1.1 (0.02)	1.1 (0.02)	1.04 (0.02)	**0.025**	0.096	0.081	0.955	0.997	0.315	0.196
GCIPL ST/IN	0.96 (0.02)	0.97 (0.02)	0.92 (0.02)	0.96 (0.02)	0.295	0.997	0.486	1.000	0.346	0.999	0.391
GCIPL ST + IT/SN + IN	0.97 (0.01)	0.94 (0.01)	0.92 (0.01)	0.96 (0.01)	**0.025**	0.342	**0.041**	0.977	0.681	0.628	0.082
GCIPL S + SN + ST/I + IN + IT	0.99 (0.02)	1.03 (0.02)	1.01 (0.02)	1 (0.02)	0.434	0.434	0.908	0.998	0.861	0.581	0.956

RNFL, retinal nerve fiber layer; GCIPL, ganglion cell–inner plexiform layer; SE: spherical error, PACG: primary angle-closure glaucoma; POAG: primary open-angle glaucoma; SOAG: secondary open-angle glaucoma; S, superior; I, inferior; SN, superonasal; IN, inferonasal; ST, superotemporal; IT, inferotemporal. *p*1 (Healthy vs. PACG); *p*2 (Healthy vs. POAG); *p*3 (Healthy vs. SOAG); *p*4 (PACG vs. POAG); *p*5 (PACG vs. SOAG); *p*6 (POAG vs. SOAG). Adjusted *p*-values were obtained using ANCOVA controlling for age and sex where appropriate. Bold *p*-values indicate statistical significance (*p* < 0.05).

**Table 3:** ROC analysis of OCT parameters for differentiating POAG and PACG.

	AUC (95% CI)	Threshold	Sensitivity	Specificity
Average RNFL (µm)	0.69 (0.58–0.79)	92.5	70.9%	64.7%
GCIPL S (µm)	0.67 (0.57–0.77)	83.5	70.9%	67.3%
GCIPL I (µm)	0.63 (0.52–0.74)	83.5	67.3%	64.2%
GCIPL IN (µm)	0.63 (0.52–0.74)	92.5	96.4%	30.2%
GCIPL SN (µm)	0.66 (0.55–0.76)	82.5	54.5%	71.7%
GCIPL ST (µm)	0.68 (0.58–0.78)	81.5	74.5%	54.7%
GCIPL IT (µm)	0.59 (0.49–0.7)	84.5	78.2%	49.1%
Average GCIPL (µm)	0.65 (0.54–0.76)	79.5	63.6%	67.9%
Minimum GCIPL (µm)	0.55 (0.44–0.66)	34.5	47.3%	67.9%
GCIPL S/I	0.52 (0.41–0.63)	1.01	54.5%	59.6%
GCIPL SN/IN	0.53 (0.42–0.64)	1.01	50.9%	66%
GCIPL ST/IT	0.63 (0.52–0.75)	0.98	66%	59.6%
GCIPL SN/IT	0.6 (0.49–0.72)	1.06	58.5%	68.1%
GCIPL ST/IN	0.58 (0.47–0.69)	0.85	25.5%	98.1%
GCIPL ST + IT/SN + IN	0.54 (0.42–0.65)	0.88	34.5%	94.3%
GCIPL S + SN + ST/I + IN + IT	0.54 (0.42–0.65)	1	49.1%	67.3%

ROC, receiver operating characteristic; OCT, optical coherence tomography; POAG, primary open-angle glaucoma; PACG, primary angle-closure glaucoma; AUC, area under the curve; CI, confidence interval; RNFL, retinal nerve fiber layer; GCIPL, ganglion cell–inner plexiform layer; S, superior; I, inferior; IN, inferonasal; SN, superonasal; ST, superotemporal; IT, inferotemporal; S/I, superior-to-inferior GCIPL ratio; SN/IN, superonasal-to-inferonasal GCIPL ratio; ST/IT, superotemporal-to-inferotemporal GCIPL ratio; SN/IT, superonasal-to-inferotemporal GCIPL ratio; ST/IN, superotemporal-to-inferonasal GCIPL ratio; ST + IT/SN + IN, temporal-to-nasal GCIPL ratio; S + SN + ST/I + IN + IT, superior-to-inferior hemispheric GCIPL ratio.

**Table 4:** ROC analysis of OCT parameters for differentiating POAG and SOAG.

	AUC (95% CI)	Threshold	Sensitivity	Specificity
Average RNFL (µm)	0.6 (0.49–0.72)	93.5	72.7%	51.1%
GCIPL S (µm)	0.65 (0.54–0.75)	82.5	67.3%	61.7%
GCIPL I (µm)	0.66 (0.56–0.77)	78.5	49.1%	78.7%
GCIPL IN (µm)	0.62 (0.51–0.73)	92.5	96.4%	29.8%
GCIPL SN (µm)	0.62 (0.51–0.73)	76.5	34.5%	87.2%
GCIPL ST (µm)	0.66 (0.55–0.77)	81.5	74.5%	51.1%
GCIPL IT (µm)	0.64 (0.53–0.74)	85.5	80%	44.7%
Average GCIPL (µm)	0.64 (0.54–0.75)	77.5	54.5%	74.5%
Minimum GCIPL (µm)	0.59 (0.47–0.7)	32.5	38.2%	80%
GCIPL S/I	0.55 (0.44–0.66)	0.99	67.3%	48.9%
GCIPL SN/IN	0.54 (0.43–0.66)	1.02	45.5%	72.3%
GCIPL ST/IT	0.52 (0.41–0.64)	0.92	25.5%	89.4%
GCIPL SN/IT	0.57 (0.46–0.69)	1.11	32.7%	85.1%
GCIPL ST/IN	0.57 (0.46–0.68)	0.85	25.5%	93.6%
GCIPL ST + IT/SN + IN	0.59 (0.48–0.7)	0.88	34.5%	91.5%
GGCIPL S + SN + ST/I + IN + IT	0.55 (0.43–0.66)	1.02	40%	80.9%

ROC, receiver operating characteristic; OCT, optical coherence tomography; POAG, primary open-angle glaucoma; SOAG, secondary open-angle glaucoma; AUC, area under the curve; CI, confidence interval; RNFL, retinal nerve fiber layer, GCIPL, ganglion cell–inner plexiform layer; S, superior; I, inferior; IN, inferonasal; SN, superonasal; ST, superotemporal; IT, inferotemporal; S/I, superior-to-inferior GCIPL ratio; SN/IN, superonasal-to-inferonasal GCIPL ratio; ST/IT, superotemporal-to-inferotemporal GCIPL ratio; SN/IT, superonasal-to-inferotemporal GCIPL ratio; ST/IN, superotemporal-to-inferonasal GCIPL ratio; ST + IT/SN + IN, temporal-to-nasal GCIPL ratio; S + SN + ST/I + IN + IT, superior-to-inferior hemispheric GCIPL ratio.

**Table 5:** ROC analysis of OCT parameters for differentiating PACG and SOAG.

	AUC (95% CI)	Threshold	Sensitivity	Specificity
Average RNFL (µm)	0.58 (0.47–0.7)	86.5	76.5%	44.4%
GCIPL S (µm)	0.55 (0.44–0.67)	93.5	25%	89.4%
GCIPL I (µm)	0.5 (0.39–0.62)	96	13.2%	93.6%
GCIPL IN (µm)	0.52 (0.41–0.64)	83.5	66%	42.6%
GCIPL SN (µm)	0.57 (0.45–0.68)	93.5	32.1%	85.1%
GCIPL ST (µm)	0.54 (0.42–0.65)	89.5	30.2%	83%
GCIPL IT (µm)	0.48 (0.37–0.59)	92.5	11.3%	95.7%
Average GCIPL (µm)	0.54 (0.43–0.66)	92.5	24.5%	89.4%
Minimum GCIPL (µm)	0.47 (0.35–0.58)	42.5	43.4%	62.2%
GCIPL S/I	0.53 (0.42–0.65)	0.98	75%	38.3%
GCIPL SN/IN	0.59 (0.47–0.7)	1.01	66%	59.6%
GCIPL ST/IT	0.63 (0.52–0.75)	0.98	66%	59.6%
GCIPL SN/IT	0.6 (0.49–0.72)	1.06	58.5%	68.1%
GCIPL ST/IN	0.5 (0.39–0.62)	0.97	37.7%	74.5%
GCIPL ST + IT/SN + IN	0.58 (0.46–0.69)	0.97	83%	36.2%
GGCIPL S + SN + ST/I + IN + IT	0.6 (0.49–0.71)	1.02	38.5%	80.9%

ROC, receiver operating characteristic; OCT, optical coherence tomography; PACG, primary angle-closure glaucoma; SOAG, secondary open-angle glaucoma; AUC, area under the curve; CI, confidence interval; RNFL, retinal nerve fiber layer, GCIPL, ganglion cell–inner plexiform layer; S, superior; I, inferior; IN, inferonasal; SN, superonasal; ST, superotemporal; IT, inferotemporal; S/I, superior-to-inferior GCIPL ratio; SN/IN, superonasal-to-inferonasal GCIPL ratio; ST/IT, superotemporal-to-inferotemporal GCIPL ratio; SN/IT, superonasal-to-inferotemporal GCIPL ratio; ST/IN, superotemporal-to-inferonasal GCIPL ratio; ST + IT/SN + IN, temporal-to-nasal GCIPL ratio; S + SN + ST/I + IN + IT, superior-to-inferior hemispheric GCIPL ratio.

## Data Availability

The data presented in this study are available upon request from the corresponding author due to ethical considerations.
